# Intraoperative Evaluation of Transmitral Pressure Gradients after Edge-to-Edge Mitral Valve Repair

**DOI:** 10.1371/journal.pone.0073617

**Published:** 2013-09-02

**Authors:** Jan N. Hilberath, Holger K. Eltzschig, Stanton K. Shernan, Andrea H. Worthington, Sary F. Aranki, Martina Nowak-Machen

**Affiliations:** 1 Department of Anesthesiology, Perioperative and Pain Medicine, Brigham and Women's Hospital, Harvard Medical School, Boston, Massachusetts, United States of America; 2 Department of Anesthesiology, University of Colorado Denver, Denver, Colorado, United States of America; 3 Division of Cardiac Surgery, Brigham and Women's Hospital, Harvard Medical School, Boston, Massachusetts, United States of America; 4 Department of Anesthesiology and Intensive Care Medicine, Tübingen, Germany; University of Colorado Denver, United States of America

## Abstract

**Objective:**

Edge-to-edge repair of the mitral valve (MV) has been described as a viable option used for the surgical management of mitral regurgitation (MR). Based on the significant changes in MV geometry associated with this technique, we hypothesized that edge-to-edge MV repairs are associated with higher intraoperative transmitral pressure gradients (TMPG) compared to conventional methods.

**Methods:**

Patient records and intraoperative transesophageal echocardiography (TEE) examinations of 552 consecutive patients undergoing MV repair at a single institution over a three year period were assessed. After separation from cardiopulmonary bypass (CPB), peak and mean TMPG were recorded for each patient and subsequently analyzed.

**Results:**

84 patients (15%) underwent edge-to-edge MV repair. Peak and mean TMPG were significantly higher compared to gradients in patients undergoing conventional repairs: 10.7±0.5 mmHg vs 7.1±0.2 mmHg; P<0.0001 and 4.3±0.2 mmHg vs 2.8±0.1 mmHg; P<0.0001. Only patients with mean TMPG ≥7 mmHg (n = 9) required prompt reoperation for iatrogenic mitral stenosis (MS). No differences in peak and mean TMPG were observed among edge-to-edge repairs performed in isolation, compared to those performed in combination with annuloplasty: 11.0±0.7 mmHg vs 10.3±0.6 mmHg and 4.4±0.3 mmHg vs 4.3±0.3 mmHg. There were no differences in TMPG between various types of annuloplasty techniques used in combination with the edge-to-edge repairs.

**Conclusions:**

Edge-to-edge MV repairs are associated with higher intraoperative peak and mean TMPG after separation from CPB compared to conventional repair techniques. Unless gradients are severely elevated, these findings are not necessarily suggestive of iatrogenic MS. Thus, in the immediate postoperative period mildly elevated TMPG can be expected and tolerated after edge-to-edge mitral repairs.

## Introduction

Cardiac surgical intervention for the treatment of significant mitral regurgitation (MR) currently involves a wide variety of techniques. Over the last two decades there has been a shift in surgical management with increasing emphasis on mitral valve (MV) repair over replacement due to purported advantageous patient outcomes [1], [2].

**Figure 1:**
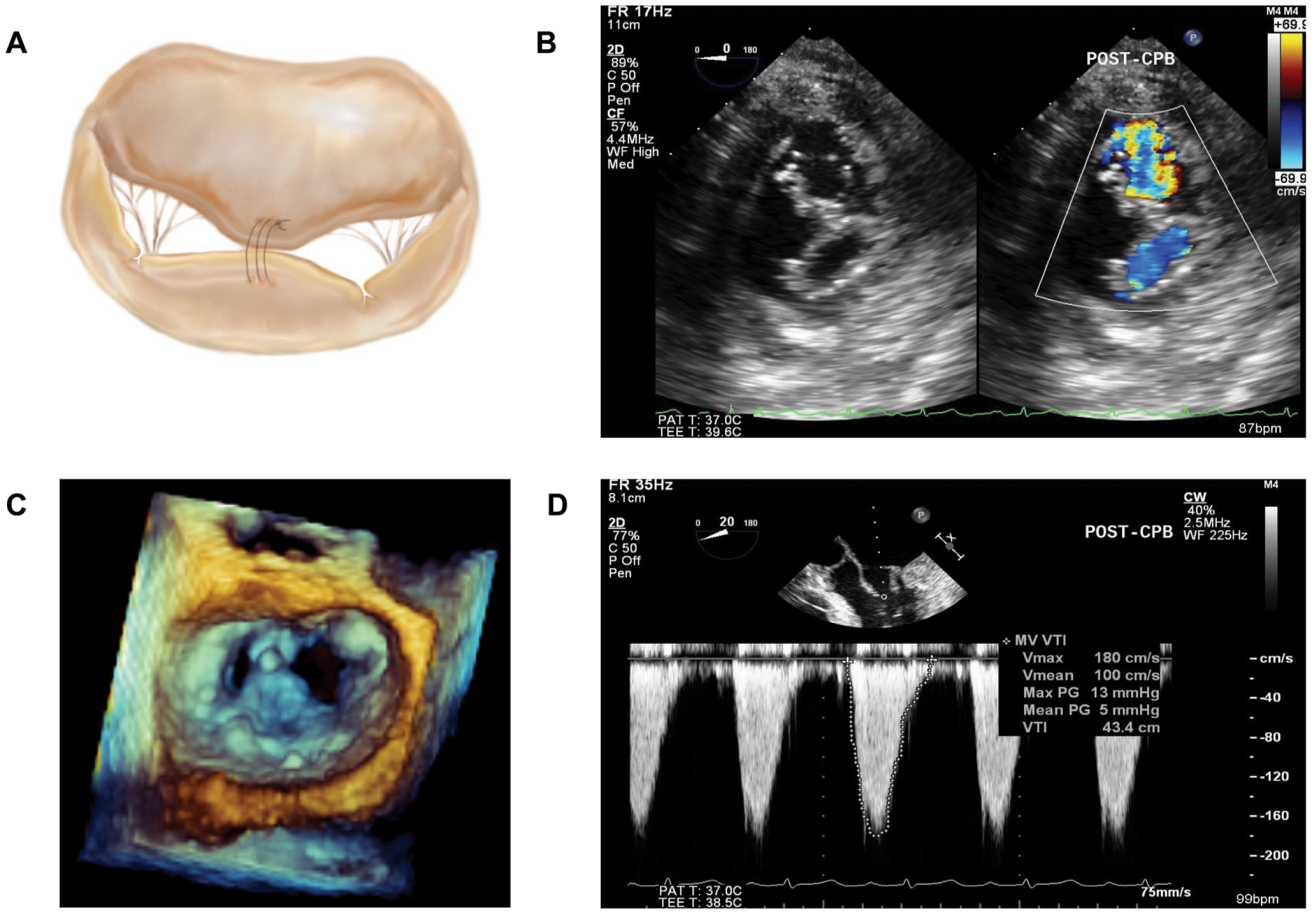
Edge-to-edge repair of the mitral valve. (A) Drawing of a central edge-to-edge (Alfieri) repair shown from the surgeons' perspective. (B) Two dimensional transesophageal echocardiographic (TEE), transgastric short axis view after edge-to-edge repair highlighting the double-orifice geometry in B-mode and Color Doppler echocardiography. (C) Still image of the mitral valve after edge-to-edge repair captured from a three-dimensional TEE, full volume data set shown en-face from the left atrial perspective. (D) Measurement of peak and mean transmitral mitral pressure gradient (TMPG) obtained with continuous-wave Doppler from the midesophageal four-chamber view following an edge-to-edge repair.

The edge-to-edge surgical technique, also referred to as ‘Alfieri stitch’, was introduced in the early 1990's [3]. This technique involves the creation of a double orifice by approximating the free edges of the anterior and posterior mitral leaflet at the non-coaptation site of the regurgitant jet without producing clinically significant mitral stenosis (MS) (Figure 1). In cases of more eccentric regurgitant lesions, an asymmetric Alfieri stitch can be employed to create a paracommissural single orifice, leaving the MV with a smaller valve area. The Alfieri stitch has gained widespread acceptance as a repair technique for various etiologies of MR [4], [5], [6], [7], and has also been adopted as a salvage option for suboptimal conventional repairs or to prevent systolic anterior leaflet motion [8]. Usually, the edge-to-edge repair is combined with a MV ring annuloplasty to stabilize the repair and either eliminate more complex or multiple regurgitant jets, or to correct dilated MV annular structures associated with functional MR [9], [10].

An important concern during MV repair is the potential for acute, iatrogenic MS. The estimation of diastolic pressure gradients conventionally derived from a transmitral flow velocity spectral Doppler profile using the simplified Bernoulli equation correlates well with invasive measurements, and is considered a Level 1 recommendation in assessing the stenotic MV [11]. In a recent study, we examined the use of intraoperative TEE as a modality to diagnose iatrogenic MS immediately after MV repair, and demonstrated that a peak transmitral pressure gradient (TMPG) ≥17 mmHg or mean TMPG ≥7 mmHg reproducibly predicted clinically relevant MS requiring timely reoperation [12]. We also found that intraoperatively acquired pressure half time (PHT) varied considerably in surgical patients between various MV repair techniques, and was therefore not useful in predicting the need for surgical re-exploration. The Alfieri stitch inherently results in significant changes in MV anatomy by creating a double orifice valve with reduced leaflet mobility. Consequently, questions of durability, subsequent valve function and exercise reserve have been proposed in the literature [13], [14], [15]. However, it remains unclear if mitral edge-to-edge repair leads to elevated TMPG intraoperatively compared to conventional repair, and if the incidence of immediate postoperative MS is increased in this patient population. Therefore, we analyzed our previous data [12] to first determine differences in postoperative TMPG between patients with edge-to-edge repair and patients undergoing conventional MV repairs. Secondly, we aimed to define ‘allowable’ or ‘expected’ intraoperative TMPG for edge-to-edge MV repairs to facilitate intraoperative communication regarding surgical and procedural decision-making, and also to be able to gauge the need for timely re-intervention for iatrogenic MS.

## Methods

### Patient Population

552 consecutive patients who underwent MV repair for MR over a three-year period at the Brigham and Women's Hospital, Boston and who had intraoperative, post-CPB transmitral Doppler flow velocity profiles recorded during routinely performed TEE were included in this analysis. Data were collected prospectively as part of a Brigham and Women's Hospital and Partners Human Research Committee–approved protocol, with a waiver of informed consent to review the patients' medical records. The records were reviewed for patient demographics, type of surgical procedure, type of MV repair, intraoperative TEE examination data, and incidence and indication for MV reoperation prior to hospital discharge.

### Transesophageal Echocardiography

Comprehensive routinely performed intraoperative TEE examinations were conducted with multiplane TEE probes (Siemens, Mountain View, CA; Philips Healthcare, Inc., Andover, MA). Peak TMPG were calculated using the simplified Bernoulli equation and derived mean TMPG were obtained from transmitral diastolic Doppler flow velocity curves by continuous-wave Doppler obtained in a midesophageal four chamber view following successful wean from CPB. TEE examinations were electronically recorded and analyzed off-line by two physicians with extensive experience in perioperative TEE. Peak and mean TMPG were obtained from the average of separate measurements of post-CPB transmitral Doppler flow velocity profiles obtained over three consecutive heart beats. As PHT measurements have been previously shown to greatly vary within this patient population, this parameter was omitted from the analysis [12], [16].

### Decision to surgically revise the initial mitral valve repair

As previously described, the decision to reoperate was made on a case-by-case basis weighing operative considerations and individual patient factors [12]. Intraoperative echocardiographic findings of concern (e.g., leaflet restriction) were discussed with the surgical team by the cardiac anesthesiologists who performed the TEE examination. While intraoperative echocardiographic measurements of TMPG were immediately available, cut-off values indicating significant acute MS following repair were not known at the time of data collection for this study.

### Statistical Analysis

Demographics were tabulated and descriptive statistics computed. Numerical results are expressed as mean ±SEM. The echocardiographic data from the two independent analyses were averaged. Mean values for echocardiographic parameters were analyzed by unpaired t-test with the use of Graph Pad Prism 5 software for Windows (San Diego, CA, USA). When numerical P values are not specified, P<0.05 was considered significant.

## Results

### Patients

552 consecutive patients who underwent MV repair and had interpretable Doppler exams were included in this analysis. An additional 26 patients were excluded as they had insufficient TEE recordings to measure TMPG. There were no demographic and preoperative differences found between Alfieri and non-Alfieri repairs with a male-to-female patient ratio of approximately 2∶1. The majority of patients (>75%) in both groups underwent mitral repairs alone or in combination with CABG (Table 1). 84 (15%) patients underwent edge-to-edge repairs as part of their surgical procedure: 52 Alfieri repairs were combined with annuloplasty, while 29 patients underwent isolated Alfieri repairs. Preferred annuloplasty prosthetics included Cosgrove-Edwards bands (n = 341) or Carpentier-Edwards rings (n = 181) (Table 2).

**Table 1:** Patient characteristics (n = 552).

	Edge-to-edge (n = 84)	Conventional (n = 468)
Age (yr±SD)	61.4±14.8	63.8±3.7
Gender (F/M)	29/55	158/310
EF (%±SD) (pre/post)	50±15/49±14	49±14/49±13

Surgical Procedure	Edge-to-edge		Conventional	
	Primary	Reoperation	Primary	Reoperation
MV Repair	39	1	164	9
MV Repair + CABG	23	1	203	19
MV Repair + Other Valve (AVR, TVP)	8	2	37	13
MV Repair + Other Valve (AVR, TVP) + CABG	10	0	23	0

yr: years; SD: standard deviation; F/M: female/male; EF: left ventricular ejection fraction by TEE exam; pre/post: before/after cardiopulmonary bypass; CABG: coronary artery bypass grafting, MV: mitral valve, AVR: aortic valve replacement, TVP: tricuspid valve repair.

**Table 2:** Surgical details of mitral valve repairs.

Edge-to-edge (n = 84)		Conventional (n = 468)	
Alfieri + Annuloplasty	49	Isolated Annuloplasty	453
Isolated Alfieri	29	Annuloplasty + Leaflet Resection	6
Alfieri + Leaflet Resection	1	Annuloplasty + Pericardial Patch	1
Alfieri + Commisurotomy	1	Annuloplasty + Maze procedure	1
Alfieri + Chordal Repair	1	Annuloplasty + Commisurotomy	1
Alfieri + Annuloplasty + Leaflet Resection	3	Annuloplasty + Chordal Repair	1
		Other	5

Annuloplasty systems	Edge-to-edge	Conventional
Cosgrove-Edwards (n = 314)	37	277
Carpentier-Edwards (n = 181)	12	169
Carbomedics (n = 12)	2	10
Duran (n = 7)	1	6
Medtronic (n = 1)	0	1

### Transmitral pressure gradients in edge-to-edge repairs

**Figure 2:**
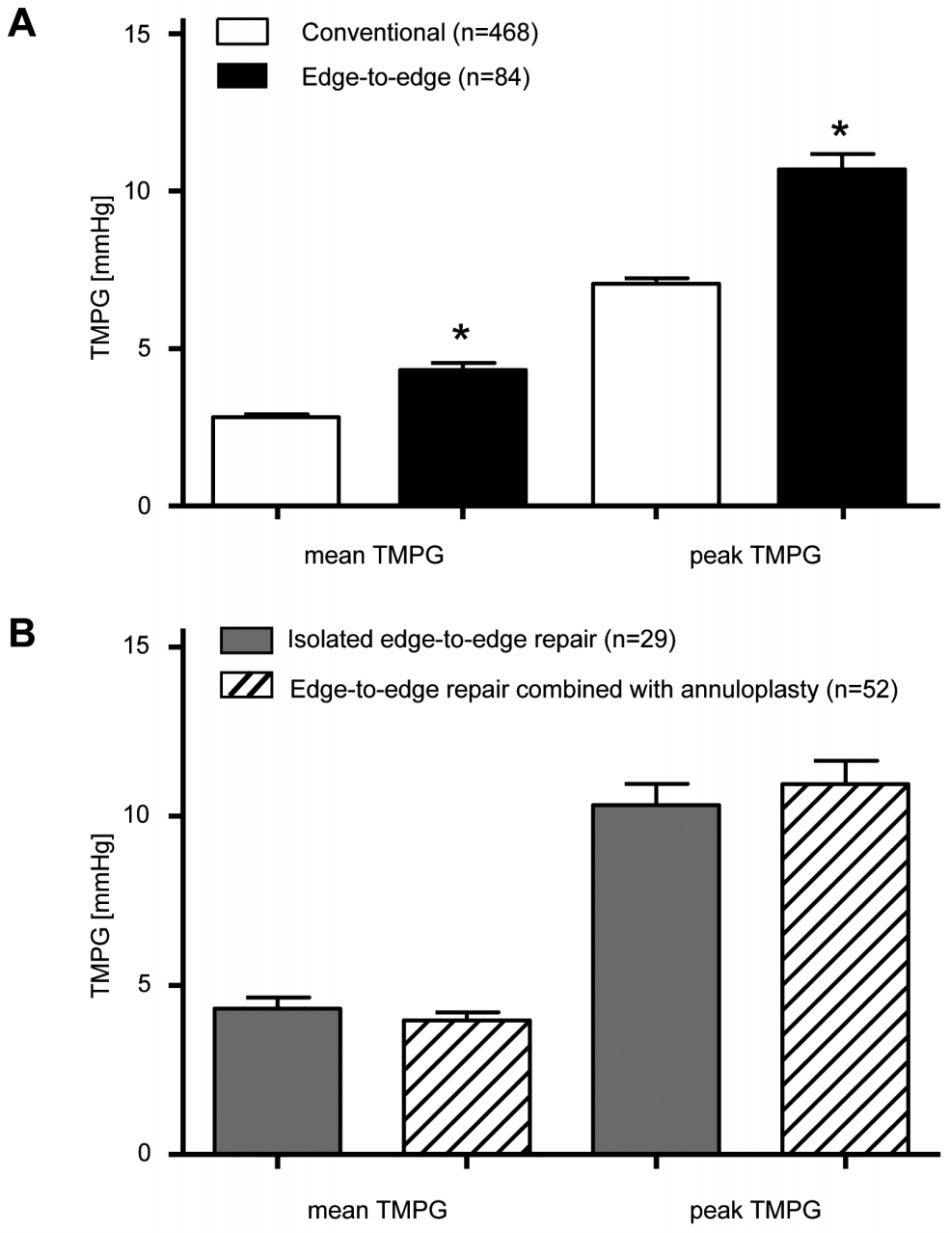
Transmitral Pressure Gradients in edge-to-edge versus conventional mitral valve repairs. (A) Transmitral mitral pressure gradients (TMPG) were determined after separation from cardiopulmonary bypass and are shown for all patients after edge-to-edge and conventional mitral valve repairs. Values represent the mean ±SEM for n = 84. *P<0.0001 vs. conventional repair. (B) TMPG after edge-to-edge repair were performed in isolation (n = 29) or in combination with an annuloplasty ring (n = 52). There were no differences in TMPG with the addition of an annuloplasty system.

Inter- and intraobserver variability of gradient measurements were minimal with Pearson's r and 95% CI of 0.989 (0.987, 0.991) for peak TMPG and 0.964 (0.958, 0.970) for mean TMPG. Patients in the edge-to-edge repair group had mean and peak TMPG of 4.3±0.2 mmHg and 10.7±0.5 mmHg, respectively. These Doppler-derived gradients were significantly higher than mean and peak TMPG in patients following conventional repairs: 2.8±0.1 mmHg and 7.1±0.2 mmHg, P<0.0001 (Figure 2A). There were no differences in TMPG in patients in whom edge-to-edge repair was combined with annuloplasty vs no annuloplasty: mean 4.3±0.3 mmHg vs 4.3±0.3 mmHg and peak 10.3±0.6 mmHg vs 11±0.7 mmHg, respectively (Figure 2B). Mean and peak TMPG were also not influenced by the various types of annuloplasty rings in the conventional repair group.

### Incidence of mitral stenosis in edge-to-edge versus conventional repairs

TMPG measurements reliably predicted the occurrence of iatrogenic MS requiring prompt reoperation in our previous study [12], however this initial investigation did not include a specific analysis by type of MV repair. Four of nine patients requiring reoperation for postoperative iatrogenic MS had undergone edge-to-edge repair as part of their initial procedure (3 edge-to-edge repairs were combined with annuloplasty, 1 isolated Alfieri repair). The five patients in the conventional repair group had undergone MV repairs with annuloplasty systems (2 Cosgrove-Edwards, 3 Carpentier-Edwards). As previously reported, all patients with a peak TMPG of at least 17 mmHg or mean TMPG of at least 7 mmHg required prompt reoperation for significant MS after MV repair. However, no differences in average TMPG were found between edge-to-edge and conventional repair groups in the iatrogenic MS patients with: peak TMPG 22.5±3 mmHg vs 22.4±4 mmHg; mean TMPG 10.3±1.2 mmHg vs 10.8±2.9 mmHg. While the overall incidence for MS after Alfieri repairs was 4.8% (4/84) vs 1% (5/468) after conventional repairs, this study was not powered to measure significant differences in the incidence of post-repair MS between the two groups.

## Discussion

While MV repair remains the preferential technique for surgical approaches to MV disease, a wide variety of techniques have been recommended based upon the mechanism and etiology of disease, as well as surgeon and institutional preferences. The edge-to-edge or ‘Alfieri’ procedure is convenient because it can be performed efficiently, and can be applied to a variety of regurgitant lesions and is technically less demanding [17]. Nonetheless, considerable expertise is required to localize the precise site for mitral leaflet approximation and the exact extent of the suture line in order to eliminate MR, while inevitably reducing the orifice area. With increasing numbers of percutaneous MV repairs being performed, the number of patients undergoing edge-to-edge repairs will likely rise [18]. To date, there is only sparse data available to counsel the intraoperative echocardiographer regarding the ‘expected’ or ‘acceptable’ post-MV repair TMPG since the incidence of immediate iatrogenic MS after edge-to-edge repairs has not been comprehensively studied in large populations. It is also unclear how the addition of an annuloplasty prosthetic influences TMPG, compared to an isolated edge-to-edge repair. In a computational model, Redaelli et al. have shown that the absence of an annuloplasty leads to accelerated failure of the MV repair in part due to increased stress on the suture line as well as the entire valvular apparatus [19]. Alfieri et al. indicated that concomitant annuloplasty represents a key factor for the long-term durability of edge-to-edge repairs as the reduction of annular size increases the coaptation surface of the leaflets, and prevents subsequent annular dilatation [20].

Several studies have addressed the question of the hemodynamic effects of edge-to-edge repair as well as postoperative MV function and functional reserve at rest and during exercise. The hemodynamic performance of the double-orifice mitral valve seems to depend more on total valve area and cardiac output than on the change in geometry (i.e., bow-tie shape) [21]. Maisano et al. used a mathematical model to show that the flow through each valve orifice was very similar to the flow through a single-orifice of an area equal to the sum of the two orifice areas. In addition, flow velocities through the two orifices were equal, even when orifice sizes were significantly different, suggesting that Doppler sampling in either of the two orifice areas was sufficient to measure TMPG [24]. Frapier and colleagues compared conventional repair techniques with edge-to-edge repairs and performed rest and exercise transthoracic echocardiograms along with physiologic testing to measure maximal oxygen uptake [22]. Despite significantly smaller postoperative MV areas (2.5 vs 2.9 cm^2^), the edge-to-edge technique did not result in higher TMPG compared to traditional Carpentier-type repairs: the mean mitral gradients at rest were not significantly different between the two groups (3.8 vs 3.3 mmHg). At peak exercise, the increase in TMPG pressure gradients and maximum oxygen uptake were also comparable between both groups. It was hence postulated that artificially created double-orifice valves still follow physiologic principles during exercise with adequate valvular reserve in response to increased cardiac output. However, the application of these findings to the intraoperative setting remains unclear.

Barring any patient-specific contraindications, TEE is a safe imaging modality that is commonly used in cardiac surgery [23] and has become a standard imaging modality for the intraoperative evaluation of MV disease including MS [24], [25]. However, echocardiographic measures for quantifying native MS may be difficult to apply intraoperatively after MV repair due to the acute changes in valve geometry and chamber compliance. Acute MS following MV surgery can present immediately after separation from CPB, but the true incidence of MS has not been comprehensively studied, as most previous publications are either case reports or represent only relatively small sample sizes [26]. Moreover, studies in different clinical settings might be influenced by differing patient characteristics. Limitations in using Doppler echocardiography to assess MS severity have been described in surgical patients, and good correlation between PHT and MV area may be difficult to consistently demonstrate immediately after mitral valvotomy [16]. PHT varied considerably and only weakly predicted a need for reoperation for MS in our previous study [12]. Mohan et al. performed dobutamine stress echocardiography in 57 ambulatory patients with known MS to show that changes in transmitral flow are associated with only small and clinically insignificant differences in planimetered MV area, but more pronounced when determined by PHT [27].These findings emphasize that PHT is dependent on hemodynamic variables other than orifice area including left atrial and ventricular compliance. Estimating MV area using the PISA (proximal isovelocity surface area) technique has been studied in patients with native MS, and can be used to calculate MR orifice area following MV surgery [28]. However, PISA has not been validated for assessing acute MS immediately after MV repair in the operating room. Furthermore, the estimation of MV area using PISA can be time consuming and impractical in the immediate post-CPB period while treating a potentially unstable patient. Finally, the assessment of MV area via planimetry has the theoretical advantage of enabling a direct measurement of orifice area, and unlike other techniques, does not depend on flow characteristics, chamber compliance or associated valve lesions. However, the intraoperative 2D visualization from a transgastric short axis view might be challenging especially after edge-to-edge repair, due to the inability to confirm exact parallel alignment to the orifice. Intraoperative 3D echocardiography is becoming increasingly more prevalent [29], [30]. The improved imaging displays and infinite cropping capabilities enabled with large volume 3D TEE data sets may overcome these shortcomings [31]. In native MS, estimation of valve area with 3D echocardiography is considered the diagnostic gold standard by some authors [32]. To date, however, no available study has examined the reliability of intraoperative 3D TEE measurements in identifying acute, iatrogenic MS immediately after MV repair. Therefore, further studies are needed to evaluate the impact of 3D TEE indices on perioperative surgical decision-making [33], [34].

TMPG obtained by Doppler echocardiography are a reliable postoperative diagnostic tool in patients with mean gradients usually <5 mmHg as determined by TTE [15]. In addition, we have previously demonstrated that intraoperative TEE-derived TMPG were highly reproducible, fairly easy to acquire, and are considered an integral part of the echo examination for the diagnosis of clinically relevant, iatrogenic MS after separation from CPB [12]. However, certain limitations of the TMPG technique are worthy of further discussion. For example, Doppler measurements can be influenced by changes in diastolic function including impaired LV relaxation and compliance. It is uncommon, though, for mean TMPG to exceed 7 mmHg due to impaired LV compliance. Furthermore Doppler echocardiography derived measures of MS may be influenced by changes in cardiac output, heart rate and associated MR [35]. Increases in cardiac output may promote MV orifice stretching and valvular reserve associated with a decrease in PHT, whereas increased flow rates can be associated with higher TMPG [36]. Cardiac output was not measured routinely in the majority of our patients because pulmonary artery catheters were rarely used in these patients, and direct quantification of cardiac output using echocardiographic techniques is time consuming and difficult in the highly volatile post-CPB period. Hence, we were unable to determine the specific influence of cardiac output on echocardiographic measures of TMPG in our patients. Nonetheless, direct and indirect echocardiographic measures of MV area appear to remain reasonably valid under conditions of varying transmitral flow [27], [36]. Given the relatively large number of patients included in this analysis and the high level of standardization of post-CPB management, we believe that our findings still remain valid for the majority of cardiac surgical patients in the operating room after MV repair. Nonetheless, only a relatively small number of patients were diagnosed with clinically significant MS in our study. Therefore, prospective studies utilizing flow dependent measures of MS severity following MV repair including a greater number of patients with iatrogenic MS may be required to determine the true incidence of intraoperative MS.

In conclusion, mean TMPG of approximately 4 mmHg and peak TMPG of approximately 10 mmHg obtained intraoperatively immediately after mitral valve edge-to-edge repairs appear to be well tolerated even though they are reproducibly higher compared to TMPG after conventional mitral valve repairs. Mean TMPG in the range of 5 to 7 mmHg may warrant a thorough discussion regarding further intraoperative management with the cardiac surgeon. The individual patient's risks and benefits of prolonging the surgical procedure to correct rather than tolerate a mild degree of intraoperative MS need to be carefully weighed. Although not pathognomonic for iatrogenic MS, patients experiencing mean gradients exceeding 7 mmHg should be monitored vigilantly in the postoperative period, and remain at the highest risk for requiring a surgical correction independent of the previous repair type. Given the potential advantages of direct orifice area measurement and its independence from transmitral blood flow, improvements in two- and three-dimensional TEE may further facilitate the diagnosis of intraoperative mitral pathologies.
